# Chronic subdural hematoma with mild to moderate symptoms: The effect of initial treatment approach on clinical outcome

**DOI:** 10.1016/j.bas.2025.104219

**Published:** 2025-02-20

**Authors:** Merijn Foppen, Roger Lodewijkx, Mariam Slot, William P. Vandertop, Dagmar Verbaan

**Affiliations:** a Department of Neurosurgery, Amsterdam UMC, University of Amsterdam, Meibergdreef 9, Amsterdam, the Netherlands; b Amsterdam Neuroscience, Neurovascular Disorders, Amsterdam, the Netherlands

**Keywords:** Humans, Hematoma, Subdural, Chronic, Retrospective studies, Conservative treatment, Drainage, Propensity score

## Abstract

**Background:**

The effect of a conservative (wait-and-watch) approach in chronic subdural hematoma (cSDH) patients with mild to moderate symptoms, is poorly studied. Surgical evacuation is effective, but inherently carries the risk of surgical or anesthetic complications.

**Research question:**

To assess the effect of conservative or operative (burrhole craniostomy) treatment on clinical outcome, in cSDH patients with mild to moderate symptoms.

**Methods:**

This single center, retrospective cohort study included 444 cSDH patients with a Markwalder Grading Scale score 1 or 2, treated between 2012 and 2022. The primary outcomes were complication rate, length of hospital stay and 30-days’ mortality. The results were analyzed using both intention-to-treat and as-treated approaches. Propensity score techniques were applied to adjust for clinical and radiological baseline differences.

**Results:**

Of the 114 conservatively treated patients, 49 (43%) crossed-over to surgery. The 330 remaining patients were treated surgically. In the intention-to-treat and as-treated analysis, initial surgery was associated with a higher complication rate (OR 2.02, 95% CI 1.04–3.94; OR 2.87, 95% CI 1.04–7.91) and longer hospital stay (β 2.34, 95% CI 0.15–4.52; β 6.62, 95% CI 3.60–9.64). Conservative treatment was associated with higher 30-day mortality (as-treated OR 0.19, 95% CI 0.06–0.66, favoring surgery), but this was unrelated to cSDH.

**Conclusion:**

In this selected cohort of cSDH patients with mild to moderate symptoms, a conservative approach was associated with less complications and hospital stay. For these patients, a ‘conservative treatment first’ regimen may therefore be considered. Corroboration in a prospective cohort with neurological and functional outcomes is warranted.

## Highlights

• Conservative treatment is associated with less complications vs. initial surgery.
• Initial surgical treatment was associated with longer hospital stay.
• Findings support a "conservative-first" approach for mild-to-moderate cSDH patients.

## 1 Introduction

Surgical evacuation and drainage of chronic subdural hematoma (cSDH) is an effective procedure, especially for patients who experience severe neurological symptoms, impairment and when the hematoma exhibits significant mass effect. A conservative (wait-and-watch) strategy is common in asymptomatic patients (Markwalder Grading Scale (MGS) score 0) and a tolerable hematoma size without significant space-occupying effect. However, for patients with mild to moderate symptoms (MGS score 1–2), both operative and conservative treatment seem justified, but superiority of one over the other has not yet been established (Berghauser et al., 2013; Holl et al., 2022; Santarius et al., 2010; Soleman et al., 2017), whereas surgery exposes these patients, often elderly and frail, to potential complications, such as postoperative intracranial hemorrhage, seizures, pneumonia, pulmonary embolism or venous thrombo-embolic events (Rohde et al., 2002; Pang et al., 2015; Chen et al., 2020). Furthermore, the result of surgery is not always satisfactory since the risk of postoperative recurrence of the cSDH is 12.7% (Lodewijkx et al., 2023).

Although a recent review showed that conservative therapy can be quite beneficial, as surgery can be avoided in 60% of cases, a conservative approach could also prolong the period of impairment due to untreated symptoms during the period awaiting spontaneous resolution of the hematoma (Foppen et al., 2023). In addition, conservative therapy also comes with the risk of (sudden) neurological decline, which could possibly lead to a worse outcome. Consequently, significant practice variation exists between physicians and hospitals, and possibly even leads to unnecessary and costly surgical interventions (Berghauser et al., 2013; Holl et al., 2022; Laldjising et al., 2020).

A direct comparison between patients treated conservatively and those treated surgically is not achievable, because of the clinical and radiological disparities that inherently follow treatment assignment. Therefore, the goal of this study is to assess the effects of initial treatment strategy on clinical outcome, while adjusting for clinical and radiological features between both groups at diagnosis.

## 2 Methods

**Fig. 1:**
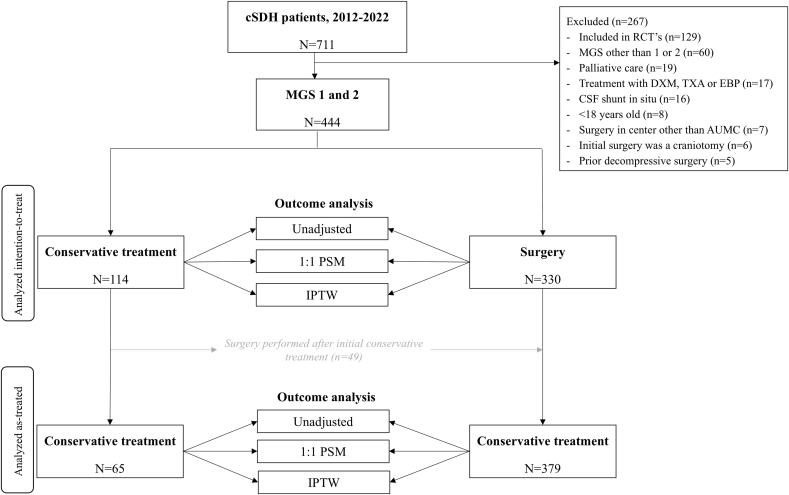
Flowchart of patient selection and analysis. Fourty-nine patients crossed-over to surgery after initial conservative treatment. These patients were considered conservatively treated in the intention-to-treat (ITT) analysis and considered as surgically treated in the as-treated (AT) analysis. In both the ITT and the AT cohort, three different methods of outcome analyzation were performed: 1) an unadjusted analysis, 2) propensity score matching (PSM), and 3) inverse probability treatment weighting (IPTW).

We performed a single-center, retrospective cohort study of all 728 patients who were identified from a retrospective registry with a cSDH between 2012 and 2022 in Amsterdam University Medical Center, a tertiary academic hospital. Patients were excluded if they met one of the following exclusion criteria: 1) a Markwalder Grading Scale (MGS) score other than 1 or 2 at diagnosis; 2) participation in a randomized controlled trial (TORCH-study or ELIMINATE-study (Foppen et al., 2019; Immenga et al., 2022)); 3) <18 years old; 4) treatment with dexamethasone, tranexamic acid, epidural blood patch or palliative care; 5) presence of a cerebrospinal fluid shunt; 6) prior decompressive surgery one year prior to diagnosis; 7) surgical technique other than burr hole craniostomy (e.g. craniotomy) and drainage; 8) history of cSDH one year prior to presentation; 9) surgery in a center other than Amsterdam UMC (see Fig. 1). The local ethics committee determined that this study did not fall under the Medical Research Involving Human Subjects Act, and a waiver for official ethical approval was obtained (waiver number: 2023.1000).

## 3 Treatment and follow-up

Treatment strategy was defined as initial conservative treatment (wait-and-watch) or initial surgical treatment (burrhole craniostomy with drainage). Due to lack of strict (inter)national guidelines, choice of treatment modality was determined by the attending neurosurgeon, based on clinical status, radiological appearance of the hematoma and mass effect. If a conservative strategy failed, due to neurological and/or radiological progression, cross-over to surgery was performed.

The standard surgical procedure was as follows: antiplatelet or anticoagulant therapy was ceased and reversed pre-operatively, if necessary. After prophylactic antibiotics (IV cefazolin), one or two burrholes per side were made under general anesthesia. After copious irrigation, a subdural or subgaleal drain was placed for 24 h. A post-operative CT-scan was only made in case of incomplete recovery, neurological deterioration or to restart anticoagulant therapy. Discharge to home, nursing home or referring hospital awaiting further placement, was based on clinical postoperative status. All surgically treated patients received a standard follow-up outpatient clinic visits six to eight weeks post-operatively. Conservatively treated patients received follow-up in the outpatient clinic with a frequency depending on neurological symptoms and radiological features. Only a minority of patients received follow-up by the referring neurologist.

## 4 Outcomes

The primary outcomes were complication rate during follow-up, length of hospital stay (LOS) and 30-days’ mortality rate. The following complications were noted: post-operative (new) intracranial hemorrhage, ischemic cerebrovascular events, postoperative wound infection or leakage, empyema or meningitis, seizures, venous thrombo-embolic events and delirium. Complication rate was a composite of any of these complications. We also conducted a separate analysis focusing solely on conservative complications. This outcome was a composite of the following complications: seizures, thrombo-embolic events and delirium. LOS stay was derived by calculating the difference between the day of admission due to cSDH and the day of discharge from the neurological or neurosurgical ward. If patients were admitted more than once, or elsewhere, the additional admitted days were also used to compute LOS. Readmissions for new diagnoses unrelated to cSDH, were not used to determine LOS.

## 5 Data collection

The following patient demographics were retrieved from patients' medical files: age, sex, medical history (arrhythmias, cerebrovascular accident (CVA), ischemic heart disease, deep vein thrombosis (DVT) or pulmonary embolism (PE), chronic obstructive pulmonary disease (COPD), malignancy, hypertension, diabetes, alcoholism, head trauma, or arachnoid cyst), use of anticoagulant or antiplatelet therapy and clinical features at diagnosis (Glasgow Coma Scale (GCS) score, headache, Markwalder Grading Scale (MGS) score and motor deficits). Motor deficit was reported as ‘yes’ if the patient experienced any form of motor function loss, independent of severity. Motor deficit was classified in more detail through the Medical Research Council scale (MRC) grade 0–5. Radiological characteristics (hematoma laterality and maximum diameter measurement in millimeters (mm), midline shift (presence and measurement in mm)) were retrieved from the radiological report. If any of these radiological variables were not described in the radiological report, these parameters were measured manually (MF and RL), as was hematoma volume using Brainlab software (Brainlab AG, Munchen, Germany). The occurrence of complications, 30-day mortality rate and the LOS was retrieved from the patients' medical files. Thirty-day mortality was coded ‘alive’ if it was clear that the patient was still living 30 days after diagnosis (e.g. from letters or notes from other specialties) or if they had deceased at a later moment. It was coded as ‘unknown’ if such information was not available.

## 6 Statistical analysis

Patient characteristics were compared using parametric and non-parametric tests. The normality of continuous variables was assessed with the Shapiro-Wilk test and were considered as normally distributed with a value > 0.9. A mean and standard deviation (SD) was calculated for normally distributed variables. A median and interquartile range (IQR) was calculated for not normally distributed variables. The differences in baseline characteristics between both groups (conservative vs. surgical treatment) were expressed in p-values by using the appropriate tests (Mann-Whitney *U* test, Chi-squared test, Fisher's exact test and independent samples *t*-test) and by calculating the standardized mean differences (SMD's)

Subsequent analyses were performed in in twofold: 1) intention-to-treat 2) an as-treated. For the intention-to-treat analysis, patients were analyzed as conservative vs. surgical treatment, without accounting for the patients that crossed over to surgery during follow-up. For the as-treated analysis, the conservatively treated patients that crossed over to surgery were transferred to the surgery group.

Multiple methods were used to assess the association of treatment strategy on the outcomes (see Fig. 1). The first method was an unadjusted analysis (binary logistic regression for the effect of surgery on complication rate and 30-day mortality, and a linear regression for LOS). Additionally, two propensity score techniques were applied to adjust for clinical and radiological baseline differences: inverse probability treatment weighting (IPTW) and propensity score matching. First, a propensity score was calculated to estimate the probability of receiving surgical treatment. The covariates that were selected for the propensity score had to have a p-value of <0.05 in univariate analyses, and a SMD >0.2 in all patients stratified by treatment strategy (see Table 1), indicating imbalance (Austin, 2011). We opted to not include baseline GCS score and presence of motor deficit at diagnosis because both variables are already incorporated in the MGS. Variance inflation factors (VIF) were calculated to check for multicollinearity. Multicollinearity was assumed when the VIF exceeded 4, which was not the case for any of the included variables.

**Table 1:** Baseline clinical and radiological characteristics of 444 patients with cSDH.

Variable	Total (n = 444)	Surgery (n = 330)	Conservative (n = 114)	p-values	SMD
Age, mean (SD)	72.5 (12)	73.2 (12)	70.3 (13)	0.032^a^	0.238
Male, n (%)	322 (73)	241 (73)	81 (71)	0.683^b^	0.044
*History*					
Arrhythmia, n (%)^1^	101 (23)	79 (24)	22 (20)	0.328^b^	0.109
Cerebrovascular accident, n (%)^1^	77 (17)	56 (17)	21 (19)	0.696^b^	0.042
Ischemic heart disease, n (%)^1^	70 (16)	51 (16)	19 (17)	0.732^b^	0.037
DVT or PE, n (%)^1^	25 (6)	14 (4)	11 (10)	0.029^b^	0.217
COPD, n (%)^1^	41 (9)	31 (9)	10 (9)	0.863^b^	0.019
Hypertension, n (%)^1^	193 (44)	143 (43)	50 (44)	0.866^b^	0.018
Malignancy, n (%)^1^	97 (22)	68 (21)	29 (26)	0.262^b^	0.120
Diabetes, n (%)^1^	124 (28)	94 (29)	30 (27)	0.692^b^	0.043
Alcoholism in history, n (%)^3^	32 (7)	23 (7)	9 (8)	0.736^b^	0.036
Head trauma, n (%)^7^	308 (71)	231 (71)	77 (69)	0.641^b^	0.051
Arachnoid cyst, n (%)	6 (1)	5 (2)	1 (1)	1.000^c^	0.059

*Medication*					
AC or AP, n (%)^1^	221 (50)	167 (51)	54 (48)	0.605^b^	0.056
Anticoagulation therapy, n (%)	112 (25)	88	24		
Antiplatelet therapy, n (%)	117 (26)	87	30		
*Clinical features at diagnosis*					
GCS median (IQR)^23^	15 (14-15)	15 (14-15)	15 (14-15)	0.110^c^	0.183
Headache, n (%)^5^	242 (55)	174 (53)	68 (61)	0.168^b^	0.152
Motor deficit, n (%)^25^	221 (53)	202 (65)	19 (18)	<0.001^b^	1.097
Pronation, drift or fall of arm/leg	64 (30)	58 (30)	6 (32)		
MRC 4	116 (54)	104 (53)	12 (63)		
MRC 3	19 (9)	19 (10)	0 (0)		
MRC 2	9 (4)	8 (4)	1 (5)		
MRC 1	2 (1)	2 (1)	0 (0)		
MRC 0	5 (2)	5 (3)	0 (0)		
Markwalder Grading Scale, n (%)				<0.001^c^	0.931
1	150 (34)	76 (23)	74 (65)		
2	294 (66)	254 (77)	40 (35)		
*Radiological characteristics*					
# Unilateral cSDH's, n (%)	285 (64)	215 (65)	70 (61)	0.472^b^^,^^a^	0.078
Left sided cSDH's (%)	150 (34)	118 (36)	32 (28)		
Right sided cSDH's (%)	135 (30)	97 (29)	38 (33)		
# Bilateral cSDH's, n (%)	159 (36)	115 (35)	44 (39)		
Midline shift, n (%)^2^	357 (81)	290 (88)	67 (59)	<0.001^b^	0.694
Midline shift in mm (SD)^3^	7.0 (5.2)	8.3 (5.0)	3.4 (3.7)	<0.001^a^	1.090
Hematoma diameter in mm (SD)^5^	19.9 (8.0)	22.1 (7.1)	13.4 (6.7)	<0.001^a^	1.247
Hematoma volume in ml (SD)^13^	118.0 (52.7)	133.0 (+-47.8)	72.0 (+-38.4)	<0.001^a^	1.408

With superscript in the column ‘variable’ is indicated for how many patients data was available if data of one or more patient(s) was missing. A drain wasn't placed in 30 patients (7.9%) who received surgery. AC: anticoagulant therapy, AP: antiplatelet therapy, cSDH: chronic subdural hematoma, IQR: interquartile range, mm: millimeters, ml: milliliters, SD: standard deviation, SMD: standardized mean difference.

Mann-Whitney *U* test.

Unilateral vs. bilateral hematoma.

a Independent *t*-test.

b Chi-squared test.

c Fishers-exact test.

For the IPTW analysis all patients were assigned a propensity score and the inverse of the weights was calculated as: 1/propensity score for the group receiving treatment (surgery), and 1/(1 - propensity score) for the group not receiving treatment (conservative therapy). Extreme weights were stabilized (Xu et al., 2010). For the propensity score matching analysis, matching was performed with the use of an optimal 1:1 matching protocol without replacement and with a caliper width equal to 0.05. Standardized differences were estimated to assess the covariate balance before and after propensity-score matching. After applying both propensity methods the effect of surgery on the primary study outcomes was evaluated with the same techniques as in the unadjusted analysis (binary logistic regression for complication rate and 30-day mortality and linear regression for LOS).

Missing values were handled through list-wise deletion.

## 7 Results

Baseline clinical and radiological characteristics of the 444 included patients are presented in Table 1. One hundred and fourteen patients (26%) received initial conservative therapy, of whom 49 (43%), crossed over to surgery (Fig. 1). See the appendix for covariate balance of the propensity methods.

Complications occurred in 79 (17.8%) patients (Table 2). In the conservative group complications occurred in 21/114 patients (18.4%) vs. 58/330 (17.6%) in the primary surgery group (p = 0.84, Table 2, Table 3). Initial surgical treatment was associated with a higher complication rate in the intention-to-treat (OR 2.02, 95% CI 1.04–3.94) and as-treated (OR 2.87, 95% CI 1.04–7.91) analysis (Table 3, Table 4). When considering only conservative complications (thrombo-embolic events, seizures and delirium), no association was found between the initial treatment strategy and these outcomes in any of the analyses.

**Table 2:** Outcomes in the total group, intention-to-treat.

Variable	Total	Surgery	Conservative
Number of cases	444	330	114
Complications, n (%)	79 (17.8)	58 (17.6)	21 (18.4)
Length of hospital stay, days (IQR)	6 (3-12)	6 (3-12)	5 (1-11)
30-day mortality, n (%)	15 (3.6)	9 (2.8)	6 (5.7)

*Complications in detail*			

Post-operative acute subdural hemorrhage, n (%)	9 (2.0)	8 (2.4)	1 (0.9)
Other intra-cranial hemorrhage, n (%)	4 (0.9)	4 (1.2)	0 (0)
Ischemic cerebrovascular event, n (%)	4 (0.9)	2 (0.6)	2 (1.8)
Wound infection or leakage, n (%)	14 (3.2)	11 (3.3)	3 (2.6)
Empyema/meningitis, n (%)	10 (2.3)	9 (2.7)	1 (0.9)
Seizure, n (%)	25 (5.6)	20 (6.1)	5 (4.4)
Thrombo-embolic event, n (%)	6 (1.4)	3 (0.9)	3 (2.6)
Delirium, n (%)	31 (7.0)	20 (6.1)	11 (9.6)

30-day mortality was unknown in 30 patients. The number of surgical cases is a composite of all patients receiving primary surgical treatment (n = 330) and patients who crossed over to surgery after initial conservative treatment (n = 49).

**Table 3:** Association of treatment strategy with outcomes, intention-to-treat.

Models	Complication rate		Conservative complication rate^b^		Length of stay (days)		30-days’ mortality^a^	
	OR (95% CI)	p-value	OR (95% CI)	p-value	β (95% CI)	p-value	OR (95% CI)	p-value
Unadjusted	0.94 (0.54–1.64)	0.84	0.76 (0.41–1.41)	0.39	1.27 (−0.88–3.42)	0.25	0.50 (0.17–1.43)	0.19
Matching	1.39 (0.55–3.49)	0.49	1.14 (0.41–3.30)	0.79	2.49 (−0.19–5.15)	0.07	0.00 (0-∞)	0.99
IPTW	2.02 (1.04–3.94)	0.04	1.79 (0.87–3.67)	0.11	2.34 (0.15–4.52)	0.04	0.45 (0.14–1.45)	0.18

*OR's and β′s reflect the effect of surgery as primary treatment strategy. Dichotomous outcomes were analyzed using logistic regression models and continuous outcomes with linear regression models*.

a Because 30-days’ mortality was unknown in 403 patients, the outcomes were analyzed in the 403 in whom the mortality rate was known. For complication rate and length of stay, propensity score matching (PSM) yielded 53 patients in both treatment groups. For the 30-day mortality, matching yielded 53 patients in both treatment groups.

b Conservative complications included only thrombo-emoblic events, seizures and delirium. IPTW, inverse probability treatment weighting.

**Table 4:** Association of surgery with main outcomes, as-treated.

Models	Complication rate		Conservative complication rate∗		Length of stay (days)		30-days’ mortality^a^	
	OR (95% CI)	p-value	OR (95% CI)	p-value	β (95% CI)	p-value	OR (95% CI)	p-value
Unadjusted	0.96 (0.49–2.05)	0.93	0.69 (0.33–1.46)	0.34	5.51 (2.81–8.20)	<0.001	0.29 (0.10–0.88)	0.03
Matching	0.68 (0.20–2.33)	0.54	0.46 (0.11–1.98)	0.30	7.95 (4.11–11.79)	<0.001	0.00 (0-∞)	0.99
IPTW	2.87 (1.04–7.91)	0.04	2.15 (0.73–6.30)	0.16	6.62 (3.60–9.64)	<0.001	0.19 (0.06–0.66)	0.005

*OR's and β′s reflect the effect of surgery as primary treatment strategy. Dichotomous outcomes were analyzed using logistic regression models and continuous outcomes with linear regression models*.

a The analyses were performed in 403 patients in whom 30-days’ mortality was known. For complication rate and length of stay, propensity score matching (PSM) yielded 43 patients in both treatment groups. For the 30-day mortality, matching yielded 36 patients in both treatment groups. ∗Conservative complications included only thrombo-emoblic events, seizures and delirium. IPTW, inverse probability treatment weighting.

The median length of hospital stay was six days (IQR 3–12, Table 2) in all patients. The median duration of hospital stay was 5 days (IQR 1–11) in the conservative group compared to six days (IQR 3–12) in the surgery group (p = 0.25, Table 2). In the as-treated analysis, primary surgical treatment was associated with a significantly longer hospital stay (p < 0.001 in all analyses, Table 5a, Table 5b). Also in the intention-to-treat IPTW analysis conservative therapy was associated with a shorter hospital stay (p = 0.04).

**Table 5a:** Mortality, intention-to-treat.

Cause 30-days’ mortality	Surgery (n = 344)∗	Conservative (n = 114)∗∗
Total (%)	9 (2.8%)	6 (5.7%)
Related to cSDH (%)^¥^	4 (1.2%)	0 (0%)
Different (%)	1 (0.3%)	4 (3.8%)
Cause	Sepsis due to aspiration pneumonia	Sepsis (n = 2)
		Respiratory insufficiency (n = 1)
		GI bleeding due to a peptic ulcer with aspiration (n = 1)
Cause unknown (%)	4 (1.2%)	2 (1.9%)

¥p = 0.576.

∗ 30-days’ mortality was known in 323 patients.

∗∗ 30-days’ mortality was known in 105 patients. GI, gastro intestinal.

**Table 5b:** Mortality, as-treated.

Cause 30-days’ mortality	Surgery (n = 379)∗	Conservative (n = 65)∗∗
Total (%)	10 (2.9%)	5 (9.3%)
Related to cSDH (%)^¥^	4 (1.1%)	0 (0%)
Different (%)	2 (0.5%)	3 (5.6%)
Cause	Sepsis due to aspiration pneumonia (n = 1)	Sepsis (n = 2)
	Respiratory insufficiency (n = 1)	GI bleeding due to a peptic ulcer with aspiration (n = 1)
Cause unknown (%)	4 (1.1%)	2 (3.7%)

^¥^p = 1.000.

∗ 30-days’ mortality was known in 348 patients.

∗∗ 30-days’ mortality was known in 54 patients. GI, gastro intestinal.

Fifteen patients (3.6%) died within 30-days after diagnosis (Table 2) and in the unadjusted analysis there was no statistical significant difference (OR 0.50, 95% CI 0.17–1.43, Table 3) between an initial conservative or surgical approach. In the unadjusted as-treated (OR 0.29, 95% CI 0.10–0.88) and IPTW analysis (OR 0.19, 95% CI 0.06–0.66), initial conservative therapy was associated with a higher 30-days’ mortality rate, however, none of the patients in the conservative group died from a cause related to their cSDH, while four patients in the surgery group (intention-to-treat p = 0.576, as-treated p = 1.000) died from a cSDH-related cause (Table 5a and Table 5b).

## 8 Discussion

This single center, retrospective cohort study shows that a conservative approach in cSDH patients with mild to moderate symptoms is associated with less complications and a shorter duration of hospital stay, compared to a surgical approach.

Determining optimal treatment strategy for cSDH remains a challenge for neurologists and neurosurgeons. Especially for patients with mild to moderate symptoms, these decisions are guided by weighing the benefits and disadvantages of both strategies (Lee, 2019). Without studies integrating both approaches, these decisions are often quite subjective, rather than evidence-based (Jehuda et al., 2014). The abundance of literature concerning the outcomes of surgical treatment for cSDH, compared to the relatively sparse studies regarding conservative treatment, suggests that neurosurgeons are instinctively quicker drawn to surgery (Henry et al., 2022). Recent surveys concerning treatment strategy for cSDH confirm this, as in approximately 75–90% of all cases a primary surgical approach is chosen (Laldjising et al., 2020; Cenic et al., 2005; Santarius et al., 2008). The absence of guidelines and clear indicators for surgery, or predictive parameters of success of conservative surgery, makes it difficult to withhold operative treatment, as surgery often rapidly alleviates symptoms. Some studies have proposed a midline shift exceeding 5 mm, or hematoma thickness of more than 10 mm as indicators for surgery (Mehta et al., 2018; Sharma et al., 2020; Greenberg, 2010; Solou et al., 2022). However, evidence-based cut-off parameters remain lacking.

Refinement of selection of patients suitable for successful conservative treatment might even be more valuable than the identification of surgical candidates, since it can prevent potential unnecessary operations. Our study shows that a conservative approach can be associated with less complications, and therefore, that a ‘conservative therapy first’ approach for patients with mild to moderate symptoms (Markwalder grade 1–2) could be considered, especially as surgery remains an option if symptoms nevertheless progress. It could be argued that delaying surgery in patients with (minor) symptoms could lead to worse outcomes. However, literature surrounding the timing of surgery for cSDH is scarce, and the few available studies show that delaying surgery does not lead to worse outcomes (Solou et al., 2022; Venturini et al., 2019; Zolfaghari et al., 2018). Moreover, in these studies there was an indication for surgery in all patients. Thus, the clinical baseline status of these patients is presumably worse than the group in which a conservative approach might also be considered. Finally, we excluded patients with a Markwalder grade 0, as these patients are generally managed conservatively. Therefore, the dilemma of treatment allocation is absent in this group. This was substantiated by the fact that an initial conservative approach was chosen for 34 out of the 35 excluded patients with a Markwalder grade of 0.

Another reason why surgeons tend to prefer a surgical approach over a conservative approach, could be the assumption that surgery results in shorter hospitalization (Laldjising et al., 2020; Cenic et al., 2005; Santarius et al., 2008). Our study contradicts this, aligning with a recent study where the mean LOS for non-operatively treated patients (n = 223) was zero days (range 0–4), compared to three days (range 1–33) for operatively treated patients (n = 978) (Rauhala et al., 2020). Longer LOS is the most important factor accruing costs in cSDH care (Frontera et al., 2011). In addition, hospital admission and longer stay potentially also lead to an increased risk in hospital-associated complications, such as delirium, infections and functional decline (Creditor, 1993). Thus, from an economic point of view it may also be argued that a primary conservative approach is sometimes favorable over a surgical approach.

Although the 30-days’ mortality rate was lower in patients receiving immediate surgery, this does not imply that a surgical approach reduces cSDH-related mortality, since none of the deaths in the conservative group were cSDH-related, whereas four patients in the surgery group died from a cause related to their cSDH. Future studies are warranted to determine whether treatment strategy really affects cSDH-related mortality.

The ITT-analysis, analyzed the outcomes of all 114 patients who received conservative treatment, irrespective of patients that crossed over surgery. This mirrors a situation that closely aligns with the typical progression of events in clinical practice. For a more precise evaluation of the association of treatment strategy with clinical outcome, the AT-analysis was conducted, in which patients who ultimately underwent surgery were categorized under operative treatment. In our study, crossover to surgery occurred in 49 of 114 (42%) patients, leaving 65 patients in the conservative group for the AT-analysis. The interpretation of the results may have been complicated by the inclusion of both intention-to-treat and as-treated analyses. However, these analyses were necessary, as it is important to acknowledge that a crossover to surgery after initial conservative management occurs in approximately 20–40% of conservatively managed patients, making this a realistic clinical scenario (Foppen et al., 2023; Khan et al., 2024).

### 8.1 Limitations and strengths

Due to the retrospective nature of this study, neurological or functional outcomes such as the modified Rankin scale (mRs), Glasgow Outcome Scale (GOS) or modified National Institutes of Health Stroke Scale (mNIHSS), could not be assessed reliably, since these outcomes were not systematically reported in the medical files in a standardized fashion. Future studies must therefore, prospectively evaluate the effect of treatment strategy on neurological and general functioning for a more in-depth understanding of treatment benefit in cSDH. Moreover, the decision to initiate a conservative or surgical approach, and when to proceed with surgery after conservative treatment, was made by the attending neurosurgeon. This methods mirrors clinical practice, though in the absence of definitive indicators or predefined criteria, such decisions are inevitably influenced by the surgeon's subjective preferences. It is probable that future studies will face ongoing challenges in applying ambiguous, predefined criteria, given the heterogeneity of cSDH. Therefore, future studies are likely to yield greater benefits by quantifying surgeons' preferences, rather than focusing solely on the application of predefined criteria.

The main strength of this study is that the effect of both treatments was investigated in several clinical and methodological ways, and that it was corrected for confounding by indication. Nevertheless, it is not possible to completely avoid bias and the results of the propensity score methods did not always concur. The outcomes obtained through inverse probability treatment weighting were deemed to be the primary results, as no patients were excluded, resulting in a more representative study cohort compared to matching. Unobserved parameters which could have influenced treatment assignment, remain, such as the level of impairment that patients experience due to their cSDH, or clinical and radiological change over time (Austin, 2011). When symptoms develop and progress in the span of a couple of days, rather than weeks or months, neurosurgeons are intuitively more inclined to perform surgical evacuation instead of following a conservative treatment. These factors are not, or minimally, embodied by the Markwalder Grading Scale, which was used in this study (Markwalder et al., 1981). Nevertheless, this scale is currently the most important prognostic instrument for cSDH, but was initially developed for patients treated with surgery and not for patients treated with conservative treatment. Thus, it is questionable whether use of the MGS for conservatively treated patients is appropriate, or if a different or modified scale is required, integrating factors such as level of impairment and clinical or radiological change.

## 9 Conclusion

In this selected cohort of cSDH patients with mild to moderate symptoms, a conservative approach was associated with less complications and a shorter hospital stay compared to surgery, after adjusting for clinical and radiological baseline differences. A ‘conservative treatment first’ regimen, in patients with mild to moderate symptoms and a hematoma deemed radiologically appropriate for conservative management, may therefore be considered. Corroboration in a prospective cohort with neurological and functional outcomes is warranted.

## Declaration of competing interest

The authors declare that they have no known competing financial interests or personal relationships that could have appeared to influence the work reported in this paper.
