# Supplementary material for: Chronic subdural hematoma with mild to moderate symptoms: The effect of initial treatment approach on clinical outcome
Source: Brain Spine. 2025 Feb 20;5:104219. doi: 10.1016/j.bas.2025.104219 (PMC11925197; doi:10.1016/j.bas.2025.104219)
Supplement: Multimedia component 1 [file mmc1.docx]

**Supplement**

**Figure 1**

**
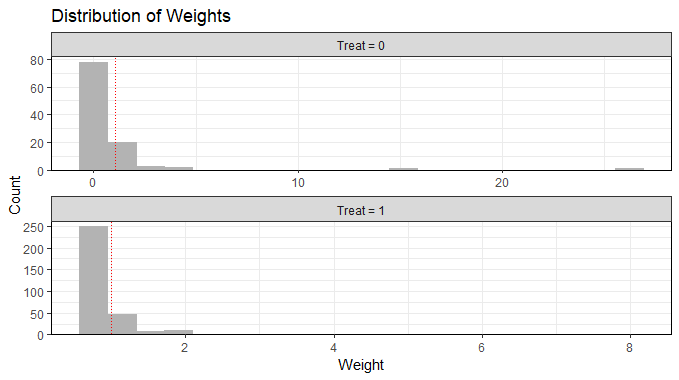
**

*Distribution of weights after applying stabilization. The group treated with initial conservative therapy equals Treat = 0 and the surgically treated group equals Treat = 1.*

**Figure 2**


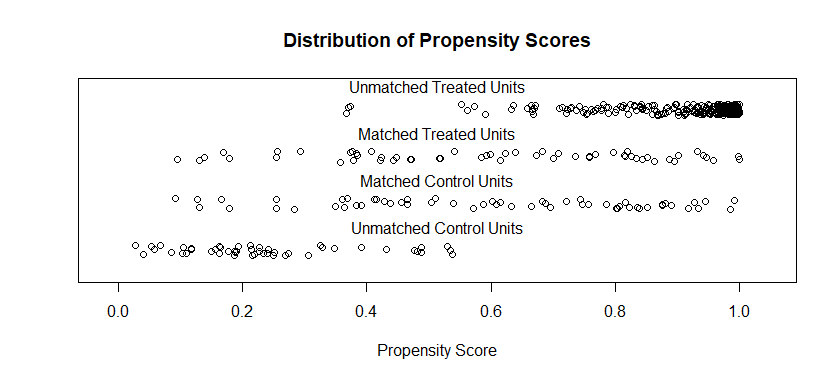


*Distribution of propensity scores. The ‘treated units’ represent the propensity scores of patients treated with initial surgical therapy. The ‘control units’ represent the propensity scores of the patients treated with initial conservative therapy.*

**Figure 3**


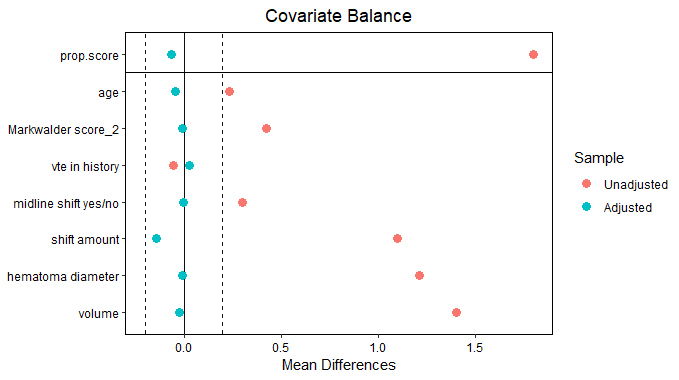


*Covariate balance, expressed in SMD after IPTW. The dotted line indicates a SMD of 0.2.*

**Figure 4**


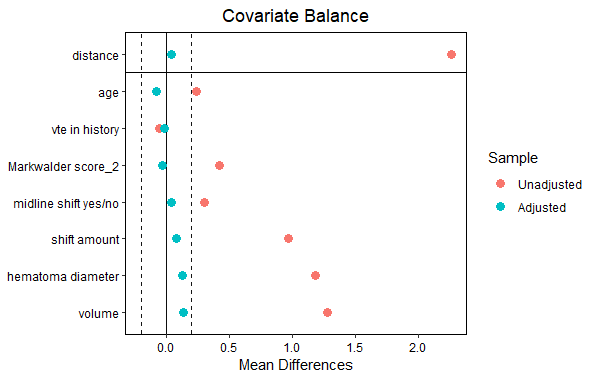


*Covariate balance, expressed in SMD after propensity score matching. The dotted line indicates a SMD of 0.2.*
